# Autophagy and Host Defense in Nontuberculous Mycobacterial Infection

**DOI:** 10.3389/fimmu.2021.728742

**Published:** 2021-09-06

**Authors:** Prashanta Silwal, In Soo Kim, Eun-Kyeong Jo

**Affiliations:** ^1^Department of Microbiology, Chungnam National University College of Medicine, Daejeon, South Korea; ^2^Infection Control Convergence Research Center, Chungnam National University College of Medicine, Daejeon, South Korea

**Keywords:** autophagy, nontuberculous mycobacteria, host defense, innate immunity, infection

## Abstract

Autophagy is critically involved in host defense pathways through targeting and elimination of numerous pathogens *via* autophagic machinery. Nontuberculous mycobacteria (NTMs) are ubiquitous microbes, have become increasingly prevalent, and are emerging as clinically important strains due to drug-resistant issues. Compared to *Mycobacterium tuberculosis* (Mtb), the causal pathogen for human tuberculosis, the roles of autophagy remain largely uncharacterized in the context of a variety of NTM infections. Compelling evidence suggests that host autophagy activation plays an essential role in the enhancement of antimicrobial immune responses and controlling pathological inflammation against various NTM infections. As similar to Mtb, it is believed that NTM bacteria evolve multiple strategies to manipulate and hijack host autophagy pathways. Despite this, we are just beginning to understand the molecular mechanisms underlying the crosstalk between pathogen and the host autophagy system in a battle with NTM bacteria. In this review, we will explore the function of autophagy, which is involved in shaping host–pathogen interaction and disease outcomes during NTM infections. These efforts will lead to the development of autophagy-based host-directed therapeutics against NTM infection.

## Introduction

Around 200 species of nontuberculous mycobacteria (NTMs) have been identified as the causal pathogens of pulmonary and ulcerative human diseases in both immunocompromised and immunocompetent subjects. The *Mycobacterium avium* complex (MAC) group including *M. intracellulare*, *M. avium* subsp. *hominissuis*, and *M. intracellulare* subsp. *chimaera* are the most common causes of NTM pulmonary diseases (NTM-PD), which are more emerging (1–3). *Mycobacteroides abscessus* (Mabc) is another frequently encountered pathogen that causes NTM-PD (4–6). The prevalence and incidence of NTM infections are increasing worldwide, and the risk of antibiotics resistance is often challenging and complex in the treatment of NTM diseases (7). Despite this, we have a lack of understanding of the virulence factors and host–pathogen interactions in terms of NTM infection.

Autophagy is an intracellular process for the maintenance of homeostasis upon stress conditions through lysosomal degradation of cytoplasmic cargos (8, 9). During a variety of infections, autophagy plays a cell-autonomous and/or a non-autonomous function to protect the hosts from infectious hazards and harmful inflammation (10). Recent studies highlighted multiple layered crosstalks of autophagy with several important processes, including innate immunity, immunometabolism, and mitochondrial function, to prevent harmful inflammation and to augment host protective function (10, 11). Therefore, autophagy-activating strategies are becoming promising not only for the development of host-directed therapeutics but also for the design of potential vaccines against mycobacterial infection (3). However, intracellular pathogens are able to develop sophisticated strategies of exploitation and subvert autophagy in order to enhance their survival in the host cells (12–14). Compared with *Mycobacterium tuberculosis* (Mtb), an extensively studied pathogen, much less is known about the function of autophagy pathways against NTM infection. In addition, the individual picture of NTM interaction with host autophagy machinery and how each NTM escapes from host autophagic responses remain uncharacterized. In this review, we focus on the recent progress of our understanding of autophagy functions in the context of host defense against NTM infections.

## Overview of Host–Pathogen Interaction During NTM Infections

NTM bacteria are diverse species that grow in the environment and are opportunistic pathogens that cause a wide spectrum of diseases in humans. The prevalence, morbidity, and mortality of NTM diseases are increasing worldwide, particularly in developed countries, associated with several predisposing factors such as aging, immunosuppressive therapy or conditions, and relevant comorbidity with chronic pulmonary diseases (15–21). NTM-PD is common in immunocompetent persons, whereas immunocompromised patients primarily suffer from disseminated diseases (16, 18). The most important human pathogens causing NTM-PD include MAC, Mabc, and *Mycobacterium kansasii*. In addition, the infections caused by NTMs also vary by geographic distribution (22, 23). NTM-PD also can be organized into clinical phenotypes (24). For example, “Lady Windermere’s syndrome” usually occurs in elderly females with a fibronodular radiographic pattern of NTM-PD (25, 26). Besides NTM-PD, NTM causes extrapulmonary diseases, including skin and soft-tissue infections, musculoskeletal infections, lymphadenitis, and disseminated disease (27, 28). Importantly, NTM treatment is often toxic and difficult because of intrinsic multidrug resistance, limited treatment options, and lengthy duration (24, 29, 30).

After infection, NTMs are found in different types of cells but extensively studied in macrophages as the primary host cells where a vast number of NTMs are able to arrest phagosomal maturation and persist, form biofilms, and even replicate (31–34). Thus, innate immune signaling activated by numerous pathogen-associated molecular patterns may contribute to host immune defense against NTM infection (32). Although the exact nature of host protective factors is uncertain, it has been long thought that T helper 1 (Th1) responses induced by interferon (IFN)-γ and interleukin (IL)-12 are crucial in the defense against NTM infection (31, 35). In addition, several genetic factors, including cystic fibrosis transmembrane conductance regulator mutations, vitamin D receptors, and polymorphisms of solute carrier 11A1 (or natural resistance-associated macrophage protein 1), are associated with NTM-PD (35, 36), although these are not specific to NTM infections. Moreover, anti-tumor necrosis factor (TNF)-α therapy during autoimmune diseases may lead to the increased risk of NTM diseases as well as tuberculosis, suggesting that TNF-α is also crucial for host defense against NTM infection (37, 38). Recent studies highlight the function of autophagy and apoptosis as another key factor for controlling mycobacteria (1, 3). In this review, we primarily discuss the current understanding of host cell autophagy in terms of host defense and controlling immunopathology during NTM infection.

## Overview of Autophagy in Terms of Mycobacterial Infections

Although this session covers a general understanding of the autophagy/xenophagy pathways and their interaction with intracellular Mtb, much uncertainty remains on the specific function of autophagy in the context of each NTM infection. In Mtb infection, there are at least three types of autophagy pathways participating in the antibacterial host defense (39). Xenophagy involves the cytoplasmic escape of Mtb through the ESAT-6 secretion (ESX)-1 system, thereby being subjected to ubiquitination system and recognized by selective cargo receptors, i.e., p62 and NDP52, for lysosomal degradation (40). Although the ESX-1 system is required for early autophagy induction, it functions in a late inhibition of autophagy flux in human primary dendritic cells (41). Xenophagy involves the core autophagy-related genes (Atg), including ULK1, Atg14, Beclin-1, and Atg5-12, which are important in the initiation of autophagosome formation and elongation step of autophagy (42). Another type of noncanonical autophagy, LC3-associated phagocytosis (LAP), involves Rubicon, NADPH oxidase 2, Beclin-1, and Atg5-12, which is also crucial for combating intracellular Mtb, which resists this process through its own effector, the LCP protein CpsA (43). In recent years, we have made considerable progress in revealing the signaling pathways that regulate xenophagy against Mtb infection. The cytosolic DNA sensor cyclic GMP-AMP synthase (cGAS)-STING signaling pathway is critically required to recognize cytosolic Mtb DNA to induce autophagy (44). A recent study showed that xenophagy could be triggered by the direct ubiquitination of Mtb surface protein Rv1468c, which contains a eukaryotic-like ubiquitin-associated domain (45). In addition, xenophagic clearance of Mtb is mediated by various E3 ubiquitin ligases, including PARK2 (46), Smurf1 *via* K48-linked ubiquitination (47), and TRIM16 through interaction with galectin-3 (48). Moreover, the lysosomal damage recognized by galectin-8 and -9 signaling promotes autophagy and antimicrobial responses against Mtb infection (49). However, it is largely unclear whether these or other signaling pathways are involved in regulating autophagy defense against NTM bacteria, which may operate different strategies compared to Mtb to survive within host cells.

So far, numerous autophagy-activating agents/drugs have been reported to enhance the activation of autophagy and phagosomal maturation through colocalization of bacterial phagosomes with autophagosomes/lysosomes (50–52). Accumulating evidence suggests that a wide range of antimicrobial strategies can be applied to promote antimicrobial activities for infectious diseases through autophagy modulation (50–53). These strategies include multiple biological pathways such as targeting selective autophagy through adaptors, regulation of posttranslational modification of key proteins, modulation of inflammatory responses, etc. (10, 52, 53).

Earlier studies showed that both *Mycolicibacterium smegmatis* and *Mycolicibacterium fortuitum* exhibit strong autophagy induction, whereas *M. kansasii* induces less induction of autophagy in macrophages (54). In addition, autophagy induced by *M. smegmatis* is independent of mTOR activity, and lipid components of *M. smegmatis* activate mTOR signaling (54). Because mTOR inhibition by rapamycin results in decreasing intracellular bacterial burden (55), simultaneous activation of both autophagy and mTOR signaling might be another immune escaping strategy manipulated by bacteria. Mabc smooth (Mabc S) variant exhibits pathogenesis mainly through the suppression of phagosomal maturation and induction of phagosome-cytosol communications. However, the Mabc rough (Mabc R) variant enhances autophagy and apoptosis and can form extracellular cords, thereby evading phagocytosis (56, 57). However, it remains largely unknown how various NTM microbes induce or suppress host cell autophagy in different tissues/cells and whether it exerts to modify host defensive system during infection. In the next session, we discuss the recent advances and perspectives on the roles of host cell autophagy in the context of infection with each NTM pathogen and explore in brief the potential autophagy-activating strategies against NTM infections.

## Autophagy in NTM Infections

### MAC and Autophagy

*M. avium* complex (MAC), among other NTMs, is the most commonly isolated species in the world (58). *M. avium* infection leads to the increase in numerous microRNAs, including miR-125a-5p that is required for autophagy activation and suppression of intracellular survival of *M. avium* in macrophages (59). MiR-125a-5p-mediated autophagy activation is induced by targeting of signal transducers and activators of transcription 3 (STAT3) in macrophages (59).

Alpha-1-antitrypsin (AAT) deficiency is closely related to the increased risk of emphysema and bronchiectasis (60), which are important predisposing factors for NTM-PD (36). Previous studies reported that AAT treatment results in the control of intracellular growth of *M. abscessus* and *M. intracellulare* in human macrophages (61, 62). Interestingly, human primary monocyte-derived macrophage culture with plasma obtained from patients with post-AAT infusion significantly increases the autophagosome formation during *M. intracellulare* infection (61). These studies may provide potential clinical significance because AAT-based, autophagy-related, adjunctive therapy could be beneficial for treatment of NTM-PD patients who have underlying diseases such as bronchiectasis along with AAT deficiency.

### *M. abscessus* and Autophagy

Mabc is the rapidly growing NTM strain and unique in the characteristics for survival inside macrophages (56). Mabc is classified into two morphotypes, i.e., S and R forms, depending on the presence of glycopeptidolipids (GPL) (56, 63–65). Mabc ESX-4 locus that encodes an ESX-4 type VII secretion system is crucial for the growth and survival within host cells through blockade of phagosomal acidification and the ability to damage phagosomes (66).

Mabc S variants reside within more intact phagosomes and are surrounded by an electron translucent zone (ETZ), whereas Mabc R variants possess a loose phagosomal membrane and lack ETZ (63). Thus, it is thought that Mabc S strains are capable of successful phagosome-cytosol communication and are more resistant to phagosomal acidification. In addition, the nature of Mabc S to favor phagosome-cytosol communication is associated with less induction of autophagy and apoptosis than those by Mabc R morphotype (63). In accordance with this, Mabc S infection of macrophages upregulates the LC3-II and p62 levels in a time-dependent manner, suggesting that Mabc S inhibits autophagic flux (67). Earlier studies suggest that the use of antibiotics such as azithromycin aggravates the impairment of autophagy during Mabc infection, thus predisposing patients with cystic fibrosis to NTM infections. Mechanistically, long-term use of macrolide drug azithromycin results in an inhibition of intracellular clearance of Mabc in human macrophages, at least due to defective autophagy and prevention of lysosomal acidification of NTM bacteria (68). Furthermore, the virulent clinical strain UC22 of Mabc, the R variant, robustly inhibits autophagic flux, thereby escaping from the clearance by host defense (69).

However, recent reports showed that treatment of the autophagy inhibitor and activator (chloroquine and rapamycin, respectively) does not affect antimycobacterial effects against Mabc R and S infection in neutrophils (70). These data suggest that autophagy is not critically involved in neutrophil antimicrobial pathways against Mabc infection. Future studies are warranted to discover the exact roles and mechanisms by which autophagy activation regulates the virulence or protective responses in different cell types and tissues during infection with Mabc and their related strains.

### *Mycobacterium marinum* and Autophagy

*M. marinum* is a natural pathogen of ectotherms to cause systemic tuberculosis-like disease and is widely used as a model organism of Mtb (71–73). *M. marinum* usually grows at 25 to 35°C and causes extrapulmonary infections at cooler surfaces like skin in humans (71, 73). The genomes of Mtb and *M. marinum* are closely related at a high degree of homology and share amino acid identity averages of 85% (72, 74).

A microscopic imaging approach through zebrafish injection of mycobacteria has lighted on tracking an *in vivo* autophagic process related to Mtb and NTM infectious diseases (75). Earlier studies reported that *M. marinum*, a model NTM for tuberculosis-like disease in zebrafish, can induce autophagosome formation *via* the ESX-1 secretion system but simultaneously actively block the autophagic flux to escape from xenophagic degradation during infection (76, 77). In *M. marinum*–infected macrophages, phagosomal escape and bacterial ubiquitination are followed by targeting the lysosome-like organelle through the autophagy-independent pathway, which does not involve atg5 or LC3 association (78). Another study showed that *M. marinum* mimG, an orthologue of Mtb Rv3242c that contains phosphoribosyltransferase, enhances intracellular bacterial survival and virulence in zebrafish. *M. marinum* mimG-induced pathogenesis is at least partly mediated due to the inhibition of autophagy in macrophages (79). Future studies are warranted to identify other bacterial effectors that alter host cell autophagy to exert immune evasion during infection. These efforts will facilitate the presentation of attractive targets for potential host-directed drug therapies during NTM infection.

*M. marinum* is targeted by selective autophagy through autophagic adaptors optineurin and p62/SQSTM1 for bacterial clearance (80). DNA damage regulated autophagy modulator 1 (DRAM1), a critical regulator of autophagy and cell death, is activated by Toll-like receptor signaling and plays an essential function in selective autophagic defense against *M. marinum* infection (81). The selective autophagy activation by DRAM1 is mediated through cytosolic DNA sensor STING and the adaptor p62/SQSTM1 (81). Indeed, DRAM1 functions through autophagic targeting and phagosomal maturation of *M. marinum*, thereby restricting bacteria during the early phase of infection. Dissemination of *M. marinum* infection is associated with defective autophagy and gasdermin Eb-mediated pyroptotic cell death in dram1 mutant zebrafish larvae (82). However, it remains to be characterized whether DRAM1 plays a crucial role in host defense to other NTM strains through activation of autophagy and prevention of cell death.

Moreover, *M. marinum* infection of microglial cells induces autophagy that can limit the intracellular replication of *M. marinum* (83). Notably, rapamycin-induced autophagy activation inhibits the intracellular survival of *M. marinum*, suggesting the role of autophagy in microglial defense against *M. marinum* (83). Because *M. marinum* is genetically closely related to Mtb in a high degree of homology (74), autophagy activation may provide a new strategy for the treatment of tubercular meningitis.

*Drosophila melanogaster* is another model host for *M. marinum* and is widely used for innate immune defense and xenophagy during mycobacterial infection (46, 84–86). In the Drosophila model, autophagy-related gene *Atg2* is required to inhibit intracellular mycobacterial growth and lipid droplets in phagocytes without changing bulk autophagy during *M. marinum* infection (85). By using atg7 mutant Drosophila, autophagy activation *in vivo* was found to contribute to antibiotic-mediated antimicrobial effects during *M. marinum* infection (87). Using unicellular eukaryote *Dictyostelium discoideum*, another model host for *M. marinum*, transcriptome analysis identified that *M. marinum* induces transcriptional activation of autophagy genes and endosomal sorting complexes required for transport (ESCRT) (88). Another study showed that the *Mycobacterium*-containing vacuole (MCV) damage induced by the ESX-1 system of *M. marinum* is recognized and repaired by the ESCRT component Tsg101, thereby leading to the containment of *M. marinum* in an intact compartment (89). In this process, autophagy and ESCRT pathways function in separate membrane repair processes in parallel for the restriction of mycobacterial growth in the cytosols during *M. marinum* infection (89). However, it is yet to be elucidated how ESCRT components are recruited to vacuolar damage sites in *D. discoideum* and whether ubiquitination system is involved in the ESCRT recruitment for membrane repair.

### *M. smegmatis* and Autophagy

Nonpathogenic *M. smegmatis* is well known to induce autophagy in macrophages through the upregulation of several autophagy-related genes and TLR2 activation. However, autophagy targeting of *M. smegmatis* is not dependent on membrane damage and ubiquitination of bacteria (90). In addition, a high dose of rapamycin treatment leads to antimicrobial activities to *M. smegmatis*, presumably due to autophagy-independent modality, because bacterial growth is also inhibited in autophagy-deficient macrophages (91). Thus, there might be an alternative mechanism by which the autophagy pathway is functional in the recognition of mycobacteria to enhance phagosomal maturation and antimicrobial responses during infection.

*M. smegmatis* infection of PC12 and C17.2 cells induces neural differentiation through an autophagy-independent pathway *via* IFN-γ and PI3K-Akt signaling pathways (92). Vitamin D3, known as a protective factor for human tuberculosis, increases intracellular *M. smegmatis* clearance and restricts host cell cytotoxicity (93). Because vitamin D3 induces the activation of antibacterial autophagy and cathelicidin to inhibit intracellular Mtb survival (94), vitamin D3-mediated *M. smegmatis* clearance is presumably mediated through autophagy and antimicrobial proteins. Future studies are warranted to clarify the roles of autophagy in antimicrobial host defense against *M. smegmatis* infection.

### *Mycobacterium ulcerans* and Autophagy

Buruli ulcer, the third most common mycobacterial disease and destructive necrotizing skin infection caused by *M. ulcerans*, is common in West and Central Africa and becoming increasingly common in southeastern Australia (95, 96). Several studies have highlighted the genetic susceptibility of *M. ulcerans* infection in the context of autophagy. Recent genetic studies showed the protective effect of the minor allele G of ATG16L1 (rs2241880) from the ulcer phenotype in Buruli ulcer (97, 98). In addition, several autophagy genes, including PRKN, NOD2, and ATG16L1, are related to susceptibility to severe Buruli ulcer (98). Importantly, the missense variant T300A (rs2241880) of the *ATG16L1* gene is associated with the development of Buruli ulcer (98). A mechanistic study showed that knock-in mice (*Atg16L1^T316A^*) harboring the human ATG16L1 variant (T300A) functions in a decrease in bacterial autophagy, thereby protective to *Citrobacter rodentium* infection through type I interferon response, similar to hypomorphic ATG16L1 mice (99). However, it is still unclear whether a certain allele of ATG16L1 (T300A) functions in the suppression of autophagy against *M. ulcerans* to confer host protection against Buruli ulcer.

A proteomics study showed that mycolactone, the potent exotoxin of *M. ulcerans*, significantly increases autophagosome formation and protein ubiquitination (100). These data strongly suggest that toxin affects host cell homeostasis, although the molecular mechanisms underlying these phenomena have not been elucidated. A genome-wide association study (GWAS) identified two variants in LncRNA genes (rs9814705 and rs76647377) in association with Buruli ulcer (97), suggesting the potential roles for LncRNAs in the pathogenesis of Buruli ulcer. Given the findings that the expression of long intergenic noncoding RNA erythroid prosurvival (lincRNA-EPS) is downregulated in primary monocytes from patients with active pulmonary tuberculosis and silencing of lincRNA-EPS enhances autophagy in macrophages during bacillus Calmette-Guérin (BCG) infection (101, 102), the LncRNA variant may play a role in the autophagy activation to modulate antimicrobial responses during Buruli ulcer further. Future studies are warranted to determine the exact role of autophagy and its related function of the identified variants of LncRNAs in Buruli ulcer. Such an effort will facilitate the development of new strategies against Buruli ulcer.

### *Mycolicibacter terrae* and Autophagy

*M. terrae* is a member of the *M. terrae* complex and slow-growing NTM and can cause antibiotic-resistant debilitating diseases, including tenosynovitis and pulmonary disease (103). Interestingly, IL-17A and IL-17F are capable of activating autophagosome formation and autophagic flux, thereby restricting the intracellular growth of *M. terrae* in RAW264.7 cells (104). The autophagic responses during several NTM infections are summarized in **Table 1**.

**Table 1:** Bacterial virulent and host defense responses in autophagy process during NTM infections.

Factors	Origin	Autophagic response	Mechanism	Study model	Ref.
***Mycobacterium avium* complex**					
** miR-125a-5p**	Host	↑	Autophagy induction by MiR-125a-5p *via* repression of STAT3 expression	THP-1 cells	(59)
***Mycobacteroides abscessus***					
** Smooth type**	Bacteria	Weak	Prevention of phagosomal maturation and acidification	BMDMs, THP-1 cells	(63)
** Smooth type**	Bacteria	↓	Upregulation of LC3-II and p62 level to inhibit autophagic flux	BMDMs	(67)
** Rough type**	Bacteria	Strong	Escapes from phagocytosis and induces more autophagy than S morphotype	BMDMs, THP-1 cells	(63)
** UC22 (R variant)**	Bacteria	↓	Increased autophagy response but inhibition of autophagic flux	RAW cells, BMDMs	(69)
***Mycobacterium marinum***					
** ESX-1**	Bacteria	↓	ESX-1 mediated induction of early autophagic responses but blockage of autophagic flux	*Dictyostelium discoideum*	(76)
** Rv3242c**	Bacteria	↓	Rv3242c-mediated inhibition of LC3-II and induction of p62 through MAPK/ERK	RAW264.7, THP-1 cells	(79)
** DRAM1**	Host	↑	Dram1-mediated p62-dependent autophagy flux and lysosomal maturation	Zebrafish, human macrophages	(81)
** ATG2**	Host	↑	Activation of JAK-STAT signaling leading to inhibition of Atg2 expression and formation of lipid droplets	Drosophila	(85)
** ESCRT**	Host	↑	Recruitment of Vps32 and Atg8 in MCVs for membrane repair	Drosophila	(89)
***Mycolicibacterium smegmatis***					
** TLR2**	Host	↑	TLR2 mediated activation of autophagy	THP-1 cells	(90)
***Mycobacterium ulcerans***					
** Mycolactone**	Bacteria	↓	Inhibition of autophagosome–lysosome fusion	L929 cells	(100)
***Mycolicibacter terrae***					
** IL-17A and IL-17F**	Host	↑	Increase in number and size of autophagosome	RAW264.7 cells	(104)

BMDM, bone marrow-derived macrophage; DRAM1, DNA damage regulated autophagy modulator 1; ESCRT, endosomal sorting complexes required for transport; ESX-1, early secreted antigenic target of 6 kDa (ESAT-6) secretion system 1; JAK-STAT, Janus kinases (JAKs), signal transducer and activator of transcription proteins; LC3, microtubule-associated protein 1 light chain 3; MAPK/ERK, mitogen-activated protein kinase/extracellular-signal-regulated kinase; MCV, Mycobacterium-containing vacuole; STAT3, signal transducer and activator of transcription 3; TLR2, Toll-like receptor 2; Vps32, vacuolar protein sorting protein 32; ↑, increase/activation; ↓, decrease/inhibition.

### A Comparative Analysis of Autophagy Among NTMs

Because different mycobacterial species have distinct virulence mechanisms for their pathogenesis, numerous NTMs and Mtb may possess differential activities and strategies to regulate host autophagy. Although there remain many gaps in the knowledge to address differential regulation of various NTMs as well as Mtb in the host defensive pathways, a recent finding reported differential immune and autophagic responses induced by Mtb and four different NTMs (Mabc, *M. smegmatis*, *M. intracellulare*, and *M. avium*) in human THP-1 cells (105). Compared to the autophagy-inducing activities by *M. smegmatis* and Mabc, the levels of autophagy induction are less in the infection with MAC and Mtb (105). Another study in RAW264.7 cells also has shown that autophagy induction by mycobacteria differs in magnitude among several species, including Mtb, BCG, and NTM (*M. smegmatis*, *M. foruitum*, and *M. kansasii*); and autophagy induction was minimal with the *M. kansasii* infection (54). Although a study suggested that long incubation of *M. kansasii* with Rapamycin could reduce the growth rate of bacteria (91), the exact role of autophagy in the regulation of *M. kansasii* infection is yet to be identified. A better understanding of the differential activities that regulate host autophagy pathways could offer a new insight for controlling a variety of mycobacterial infections.

## Autophagy-Activating Strategies For Antimicrobial Effects Against NTM Infections

Several reports have highlighted the antimicrobial roles of autophagy-activating agents against NTM. Recent studies showed that trehalose treatment results in the activation of the xenophagic flux to inhibit intracellular bacterial survival of various NTM strains as well as Mtb. Importantly, trehalose-mediated autophagy promotes the eradication of intracellular Mtb or NTMs, even in the status with co-infection with human immunodeficiency virus (HIV) (106). Trehalose-induced autophagy is mediated through the activation of TFEB, the key transcriptional factor for autophagy and lysosomal biogenesis (107), in macrophages (106). In addition, the autophagy induction by trehalose is dependent on lysosomal calcium release *via* MCOLN1 (106). These findings are corroborative with our recent data showing that the activation of nuclear receptor peroxisome proliferator-activated receptor α (PPARα) by gemfibrozil suppresses *in vitro* and *in vivo* bacterial growth of Mabc through TFEB activation (67). During Mabc infection, PPARα activation promotes nuclear translocation of TFEB and colocalization of bacterial phagosomes with lysosomes in macrophages (67).

Autophagy activation by vitamin D treatment induces autophagy to facilitate antimicrobial function through CAMP production in macrophages infected with *M. marinum* (108). In addition, the blockade of glycolysis by inhibitors such as 2-deoxyglucose (2-DG) prior to infection inhibits the proliferation of *M. marinum* in macrophages and zebrafishes (109). Thiostrepton (TSR) is an antibiotic harboring a quinaldic acid (QA) moiety that targets bacterial ribosome and induces ER stress-mediated autophagy to promote antimicrobial host defense during *M. marinum* infection (110). Similarly, rifampicin and amikacin also have antimicrobial activities against *M. marinum* in *Drosophila melanogaster* through the activation of the autophagic flux (87). Ohmyungsamycins, the cyclic peptides harboring autophagy activity, have antimicrobial activities against *M. marinum* in *D. melanogaster* (84). Moreover, autophagy activation by rapamycin exhibits a defense against *M. marinum* in microglial cells (83). These data strongly suggest that several antibiotics exhibit both direct antimicrobial and indirect host-targeting ability to enhance their effects to eliminate intracellular NTM strains. Future studies are warranted to clarify the dual mode of actions mediated by several drugs that possess potential host defense activities.

Through selective targeting intracellular pathogens, the autophagy pathway functions in the activation of antimicrobial responses, regulation of immunologic balance, and anti-inflammatory effects during infection (42, 111). Recent studies showed that the NTM-PD patients with Mabc or *Mycobacteroides abscessus* subsp. *massiliense* have pathological inflammatory responses in their peripheral blood mononuclear cells (112). In addition, resveratrol, an agonist of sirtuin 1 and 3 (113, 114), exerts a beneficial role through controlling excessive inflammation and mitochondrial homeostasis upon Mabc infection *in vivo* (115). Combined with resveratrol-induced antibacterial autophagy effects during Mtb infection (116), these data strongly suggest that autophagy-activating agents provide potential candidates for host-directed therapeutics during NTM infection. Antimicrobial responses of autophagy-activating exogenous agents against various NTM infections are summarized in **Table 2**.

**Table 2:** Antimicrobial and autophagic responses of exogenous agents against various NTM infections.

Agents	NTM	Mechanism	Study model	Ref.
**Trehalose**	*M. avium, M. fortuitum*	Induction of xenophagic flux *via* lysosomal Ca^2+^ release and TFEB activation	PBMCs, U937 and U1.1 cells	(106)
**Gemfibrozil**	*M. abscessus*	Increase in TFEB nuclear translocation	BMDMs, MDMs	(67)
**Resveratrol**	*M. abscessus*	Inhibition of inflammation by controlling mitochondrial ROS	Mice, BMDMs, Zebrafish	(115)
**Vitamin D**	*M. marinum*	Increased CAMP production and induction of autophagolysosome	THP-1, U927 and MEF cells	(108)
**2-Deoxy-D-glucose**	*M. marinum*	Increased autophagolysosome development and LC3-II	RAW264.7 cells, Zebrafish	(109)
**Thiostrepton**	*M. marinum*	Activation of PERK/eIF2a pathway mediated autophagy	RAW264.7 cells	(110)
**Ohmyungsamycins**	*M. marinum*	Activation of autophagy *via* AMPK pathway	Drosophila	(84)
**Rifampicin, Amikacin**	*M. marinum*	Increased colocalization of LC3 with lysosome	Drosophila	(87)
**Rapamycin**	*M. marinum*	Increased LC3 puncta formation	BV2 cells, Zebrafish	(83)

AMPK, 5′-adenosine monophosphate (AMP)-activated protein kinase; BMDM, bone marrow-derived macrophages; CAMP, cathelicidin antimicrobial peptide; eIF2a, eukaryotic translation initiation factor 2A; LC3, microtubule-associated protein 1 light chain 3; MDM, monocyte-derived macrophages; PBMC, peripheral blood mononuclear cell; PERK, protein kinase R-like endoplasmic reticulum kinase; ROS, reactive oxygen species; TFEB, transcription factor EB.

**Figure 1:**
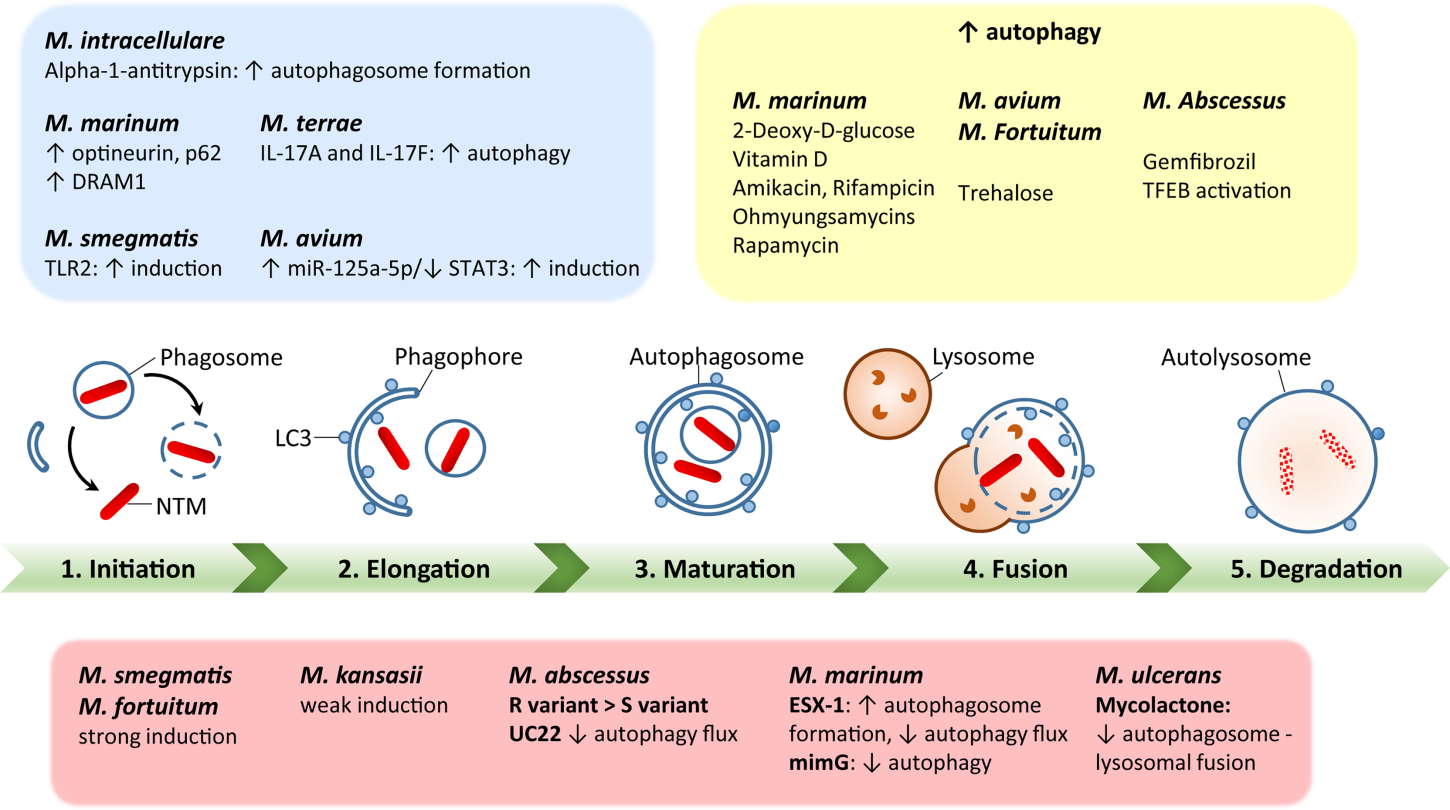
A schematic diagram of autophagy regulation by each nontuberculous mycobacterial infection. Green bar depicts the steps of autophagy, including initiation, elongation, maturation, fusion with lysosomes, and degradation. The red box summarizes each NTM response to regulate host autophagy processes. The blue box represents host factors to regulate autophagy during NTM infection. The yellow box shows the effects of autophagy-regulating agents for modulation of autophagy in the context of each NTM infection. The detailed mechanisms have been described in the text.

Additionally, BCG vaccination currently in use for immunization against Mtb could be exploited against NTM (117, 118). However, BCG vaccine interference by NTM mycobacterial species is thought to be a potential cause of its reduced efficacy against Mtb (119). In addition, several vaccine candidates with autophagy activation as a major element have been tested against Mtb in animal models (120, 121), but there are currently no recommended vaccine protocols established to study the vaccine efficacy against NTM infections. Using autophagy-related strategies to develop effective vaccinations against NTM could be a huge advance in the fight against NTM infections (3). A schematic representation of the autophagy process during several NTM infections is shown in **Figure 1**.

## Conclusion

Compared with the autophagy activation against Mtb infection, it remains to be largely uncharacterized in the roles for host autophagy/xenophagy in the context of infection caused by a variety of NTM bacteria. However, autophagy modulation seems to be a potential pathway to provide novel adjunctive therapeutics based on autophagy against various NTM infections. Future studies are warranted to understand differential roles for autophagy to regulate a complex layer of host–pathogen interaction during NTM infection.

Current knowledge is very limited on how various NTMs circumvent the autophagy process during infection. Future studies are warranted to elucidate the mechanisms by which each NTM strain induces and/or manipulates the host autophagy signaling pathway during pulmonary or extrapulmonary manifestation. Such an effort to understand autophagy functions upon NTM infection will advance the development of potential host-directed therapeutics against NTM infection.

## Author Contributions

E-KJ: designed. E-KJ, PS, and ISK: wrote and reviewed the manuscript. PS: summarized the tables. ISK: drew the figure. All authors contributed to the article and approved the submitted version.

## Funding

This work was supported by the National Research Foundation of Korea (NRF) grant funded by the Korea government (MSIT) (No. 2017R1A5A2015385) and a grant of the Korea Health Technology R&D Project through the Korea Health Industry Development Institute (KHIDI), funded by the Ministry of Health & Welfare, Republic of Korea (HI 20C0017).

## Conflict of Interest

The authors declare that the research was conducted in the absence of any commercial or financial relationships that could be construed as a potential conflict of interest.

## Publisher’s Note

All claims expressed in this article are solely those of the authors and do not necessarily represent those of their affiliated organizations, or those of the publisher, the editors and the reviewers. Any product that may be evaluated in this article, or claim that may be made by its manufacturer, is not guaranteed or endorsed by the publisher.
